# Explore the Molecular Mechanism of Apoptosis Induced by Tanshinone IIA on Activated Rat Hepatic Stellate Cells

**DOI:** 10.1155/2012/734987

**Published:** 2012-12-30

**Authors:** Tai-Long Pan, Pei-Wen Wang

**Affiliations:** ^1^School of Traditional Chinese Medicine, Chang Gung University, Kweishan, Taoyuan 333, Taiwan; ^2^Chinese Herbal Medicine Research Team, Healthy Aging Research Center, Chang Gung University, Taoyuan 333, Taiwan

## Abstract

Since the activated hepatic stellate cell (HSC) is the predominant event in the progression of liver fibrosis, selective clearance of HSC should be a potential strategy in therapy. *Salvia miltiorrhiza* roots ethanol extract (SMEE) remarkably ameliorates liver fibrogenesis in DMN-administrated rat model. Next, tanshinone IIA (Tan IIA), the major compound of SMEE, significantly inhibited rat HSC viability and led to cell apoptosis. Proteome tools elucidated that increased prohibitin is involved in cell cycle arrest under Tan IIA is the treatment while knockdown of prohibitin could attenuate Tan IIA-induced apoptosis. In addition, Tan IIA mediated translocation of C-Raf which interacted with prohibitin activating MAPK and inhibiting AKT signaling in HSC. MAPK antagonist suppressed ERK phosphorylation which was necessary for Tan IIA-induced expression of Bax and cytochrome c. PD98059 also abolished Tan IIA-modulated cleavage of PARP. Our findings suggested that Tan IIA could contribute to apoptosis of HSC by promoting ERK-Bax-caspase pathways through C-Raf/prohibitin complex.

## 1. Introduction

Hepatic fibrosis characterized by excessive deposition of extracellular matrix (ECM) proteins results from chronic liver injury due to viral, autoimmune, drug-induced, cholestatic, and metabolic diseases [1–4]. During hepatic fibrosis progresses, various cytokines and molecules including transforming growth factor-*β*1 (TGF-*β*1) and platelet derived growth factor (PDGF) would in turn stimulate hepatic stellate cells (HSCs) which then undergo phenotypic transformation from quiescent cells into proliferative and fibrogenic cells [5–8]. Activated HSCs can also mediate release of proinflammatory cytokines and tissue inhibitor of metalloproteinases (TIMP), which could cause deposition of collagen and further fibrosis [9]. With ongoing liver damage, fibrosis may progress to cirrhosis, predisposing to liver failure and hepatocellular carcinoma [10]. Since HSCs are the major cause during the progression of hepatic fibrosis, therefore, removal of HSCs by apoptosis might effectively prevent or even reverse liver fibrosis [11].

The root of *Salvia miltiorrhiza* Bunge (Labiatae), commonly known as Dan-shen, has been used as a traditional Chinese medicine for treating microcirculatory diseases in Asia [12–14]. Salvianolic acid B, a water-soluble compound in *Salvia miltiorrhiza,* has been proven to attenuate cardiac fibroblast migration, collagen, and cytokine secretion [15]. In addition, water extract of *Salvia miltiorrhiza* has attracted increasing attention due to its antifibrotic effects on dimethylnitrosamine- (DMN-) induced liver fibrosis of rat model [16, 17]. Nevertheless, it is still unknown if lipid-soluble compounds in *Salvia miltiorrhiza* could be responsible for the liver protective effect. Of the major components of *Salvia miltiorrhiza, *Tan IIA, a diterpene quinine derivative, has been reported to ameliorate oxidative stress and induce apoptosis or differentiation in various human carcinoma cell lines [18, 19]. Therefore, diterpene quinine derivatives might be potential drugs prescribed for antifibrosis due to their growth-inhibitory effects. In spite of the recent document providing the evidence that Tan IIA could induce apoptosis of rat HSC-T6 cells [20], the drug targets and underlying mechanisms remain to be explored. In this study, we evaluated the apoptotic effect and molecular mechanisms of Tan IIA upon rat HSCs.

During liver fibrosis, huge amount of proteins and molecules will be altered in quantity and quality [21]. In addition, protein quantification is a critical parameter in functional proteomics, which has the potential to reflect different stages of disease [22]. Traditional analytical methods have only provided a poor understanding of the complex underlying mechanisms or biological function. Thus, high throughput proteomics technology combined with advanced bioinformatics for data mining provides a novel pathway to large-scale screening and identifying fibrotic biomarkers and etiology [23, 24]. Our approach described here represented differentially protein profiles associated with functional “signature networks” and generated an architecture involved in protein responses to Tan IIA treatment.

With this integrated approach, we found that Tan IIA could effectively induce apoptosis of activated rat HSCs. More importantly, we have identified several protein targets that may provide novel insights into the molecular mechanisms associated with the antifibrotic efficacy of Tan IIA.

## 2. Materials and Methods

### 2.1. Materials

Specific antibodies to 14-3-3, Na^+^/K^+^-ATPase, caspase-3, -9, PARP, and COXIV were purchased from Santa Cruz (Santa Cruz, CA, USA). Polyclonal antibodies to TCTP, prohibitin, Rho-GDI, cytochrome C, GAPDH and *β*-actin were obtained from EPITOMICS (Burlingame, CA, USA). Polyclonal antibodies to Bax, bcl-2, C-Raf, AKT, phosphor-AKT, ERK (MAP kinase), and phospho-ERK were purchased from Cell Signaling (Beverly, MA, USA). PD98059 was obtained from Enzo Life Science (New York, NY, USA).

### 2.2. Preparation of SMEE and Isolation of Tan IIA

The root of * S. miltiorrhiza *(2 kg) was purchased from a traditional Chinese medicine dispensary in Taiwan and extracted with ethanol. The ethanol-extracted solution was then concentrated to give reddish syrup (290 g). The filtered and sterile extract was stored at −80°C for further animal experiments. Purification and characterization of Tan IIA have been reported in our previous paper [19].

### 2.3. Animals

Male Wistar rats weighing 200–225 g were purchased from Lasco Co. (Taiwan) and were randomly divided into three groups of three each (control: saline treated, DMN treated, and DMN/SMEE treated). In the DMN-induced fibrosis group, rats were injected intraperitoneally with DMN (10 mg/kg body weight; Sigma, St. Louis, MO, USA) for three consecutive days per week, and SMEE (10 mg/kg body weight) was orally administrated every other day for 4 weeks [16, 25]. The control group was treated with only saline. At the end of the fourth week, all of the rats were sacrificed. Their livers were excised and the specimens were immediately fixed in 10% neutral buffered formaldehyde for pathological and immunohistochemical studies. The Committee on Research Involving Animal Subjects of the Chang Gung University, Taiwan, has approved the study.

### 2.4. Histology and Immunohistochemistry

The liver tissue fixed by 5% neutral buffered formalin was immersed in paraffin and then sliced into 5 *μ*m sections. The sample slices were stained with hematoxylin-eosin (H/E) and Masson's trichrome (MT) for a histological assessment. Immunohistochemistry with *α*-SM-actin (at a 1 : 150 dilution in PBS; Neomarkers) was applied to specimens, as previously described [25, 26]. The histological changes were evaluated by using optical microscopy (Olympus BX51, Olympus Optical Co., Tokyo, Japan) in non consecutive, randomly chosen 200x or 400x histological fields. The digital photomicrographs were then processed with DP-72 (Olympus, Tokyo, Japan).

### 2.5. TUNEL Assay

TUNEL staining was performed with ApopTag Plus Peroxidase In Situ Apoptosis Detection Kit (Millipore) to evaluate the DNA damage after different treatments. Apoptosis was assessed by terminal deoxynucleotidyl transferase-mediated dUTP biotin nick end labeling (TUNEL) using 4 *μ*m thick sections according to the manufacturer's instructions. The numbers of stained and unstained cells were then counted from 5 randomly chosen fields per slide within a high-power field (×200 magnification) under a light microscope.

### 2.6. Cell Culture and MTT Assay

The immortalized rat myofibroblast cell line HSC-T6 was a kind gift of Dr. Scott L. Friedman (Mount Sinai School of Medicine, New York, NY). The HSC-T6 cells were maintained in Waymouth medium containing 10% fetal bovine serum (FBS) at 37°C in a humidified atmosphere of 5% CO_2_. HepG2 cells were cultured with DMEM medium containing 10% FBS for 24 hours (h). A total of 1 × 10^5^ cells were seeded in 24-well plates for 24 h and made quiescent by incubating in medium containing 0.2% FBS overnight. Determination of cell viability by the MTT assay was described previously after treating with various concentrations of Tan IIA for 24 h. Percent viability was calculated as (OD of drug treated sample/OD of control sample) × 100.

### 2.7. Cell Cycle Analysis under Flow Cytometry

HSC-T6 cells were treated without or with Tan IIA (at different concentration) for 24 h and fixed with ice-cold ethanol overnight. Cells were resuspended in a staining solution containing propidium iodide (PI, 50 *μ*g/mL) and DNase-free RNase (100 *μ*g/mL) at 37°C for 1 h in the dark and analyzed by a fluorescence-activated cell sorter flow cytometer (FACScalibur, Becton Dickinson, Franklin Lakes, NJ, USA). Results shown are the representative of three separate experiments.

### 2.8. Two-Dimensional Gel Electrophoresis (2-DE) and Image Analysis

The cell pellet was then solubilized in lysis buffer containing 7 M urea, 2 M thiourea, 4% CHAPS, 2% IPG buffer (Amersham Biosciences, Uppsala, Sweden), 65 mM DTT, 10 mM PMSF, and protease inhibitor cocktail (AMRESCO, Solon, OH, USA) on ice and subjected to sonication. The lysate was centrifuged at 10,000 rpm at 4°C for 30 min to remove insoluble material. The supernatant was collected and the protein was measured concentration using the Bradford assay (AMRESCO). Protein extracts (300 *μ*g) were applied to isofocusing gels and conducted at 8000 V for 90 kVh. After IEF separation and equilibration, the second electrophoresis was carried out on 10% acrylamide gels (Bio-Rad, Hercules, CA, USA) at 40 mA/gel. All gels were visualized by silver staining and then scanned using Imagescanner. Protein spots were quantified using the Prodigy SameSpots software (Nonlinear Dynamics, Newcastle, UK). Each spot intensity volume (%) was processed by background subtraction and total spot volume normalization; the resulting spot volume percentage was used for comparison between groups. More than 2.0-fold alterations at 95% confidence interval (*P* < 0.05) were considered as statistically significant [26]. All experiments were repeated 3 times from three independent experiments.

### 2.9. In-Gel Digestions and MALDI-MS/MS Analysis

Silver-stained spots were excised and in-gel digested with trypsin according to previously described procedures [26]. Briefly, the proteins were reduced with 25 mM NH_4_HCO_3_ containing 10 mM DTT and alkylated with 55 mM iodoacetamide. Then, the proteins were digested with trypsin (20 *μ*g/mL) at 37°C overnight. After digestion, the tryptic peptides were acidified with 0.5% TCA and loaded onto an MTP AnchorChip 600/384 TF (Bruker-Daltonik, Bremen, Germany). A MALDI-MS analysis was performed on an Ultraflex MALDI-TOF mass spectrometer (Bruker-Daltonik). Spectra were collected from 400 shots per spectrum over an m/z range of 600–3000 and calibrated by four-point internal calibration (m/z 956.5355, 1296.6860, 1758.9335, and 2465.198). Monoisotopic peptide masses were assigned and used for Swiss-Prot primary sequence database searches with the BioTools 2.2 software (Bruker-Daltonik) and the MASCOT search engine (Matrix Science, London, UK). Search parameters were set as follows: a maximum allowed peptide mass error of 50 ppm and consideration of 1 incomplete cleavage per peptide. The known peptide masses of keratins, internal standards, and trypsin were excluded. For MS/MS, the most intense precursor ions with a signal/noise ratio of >25 were selected after exclusion of the common background signal. The MS/MS mode was operated at 1 keV, and products of metastable decomposition at elevated laser power were detected. PMF data were acquired with close internal calibration and MS/MS data using the default instrument calibration.

### 2.10. Western Blot Analysis

Equal amount of lysated protein was separated on 12 or 15% denatured gels and transferred to membranes. After blocking, the blots were incubated with specific primary antibody overnight at 4°C and further incubated with a peroxidase-labeled anti-mice or -rabbit IgG (Santa Cruz) for 2 h. After washing in TBST, enhanced chemiluminescence (PerkinElmer, CA, USA) was used for protein detection. The band intensity was quantified using GeneTools Image Software (Syngene, UK), and GAPDH and *β*-actin were used as internal controls [25].

### 2.11. Biological Network Analysis Using MetaCore

We uploaded the differentially expressed proteins identified by the proteome tools into MetaCore software (v. 5.1 build 16271, GeneGo, St. Joseph, MI, USA) to analyze the possible networks involved in HSC-T6 apoptosis caused by Tan-IIA [27].

### 2.12. Semiquantitative RT-PCR

Total RNA was isolated from HSC-T6 cells, and single-stranded cDNA synthesis was carried out on 10 *μ*g of total RNA by a complementary DNA synthesis system for the RT-PCR according to the manufacturer's instructions (Invitrogen, USA). Primers used for the PCR experiments are listed in the following. Prohibitin: 5′-ATGGCTGCCAAAGTGTT-3′ (sense), 5′-CTGGGGGAGCTGGAGG-3′ (antisense); *β*-actin: 5′-TTGTCACCAACTGGGACGATA-3′ (sense); 5′-GATCTTGATCTTCATGGTGCT-3′ (antisense). The condition consisted of denaturing at 94° for 1 min, annealing at 53° for 1.5 min, and extending at 72° for 2.5 min. PCR was performed with 18 cycles for prohibitin and 23 cycles for *β*-actin. Each PCR product was resolved on a 1.5% agarose gel incorporated with ethidium bromide. Transcript intensities were revealed as digitalized images using a high-resolution scanner (GBOX HR; Syngene, UK) [27]. The *β*-actin transcript was used as an internal control to normalize the concentration of cDNA in each sample.

### 2.13. Gene Silencing by Small Interfering RNA

HSC-T6 cells were plated onto 6-well plates (1 × 10^5^ cells/well), maintained in antibiotic-free medium for 24 h, and transfected with a mixture containing Opti-MEM, 8 *μ*L/well Lipofectamine 2000 (Invitrogen, San Diego, CA, USA), and either 0.5 *μ*g/well scrambled siRNA (mock) or a prohibitin siRNAs (smart pool; Invitrogen) for 6 h [27]. The sequences of these siRNAs are available from the manufacturer. At 48 h after transfection, cells have been treated with or without Tan IIA and cell viability was evaluated by the MTT assay.

### 2.14. Separation of the HSC-T6 Cell Compartments

Preparation of membrane and cytoplasmic fractions was performed as described previously [28]. Briefly, the 1 × 10^8^ HSC-T6 cells with or without Tan IIA were suspended in ice-cold buffer C (CNM compartment protein extraction kit, Biochain Institute) according to the manufacturer's instructions. For cytoplasmic and mitochondrial fractions, cells were suspended in reagent A (mitochondria isolation kit for cultured cells, Thermo Scientific) which according to the manufacturer's instructions. Finally, mitochondrial, membrane, and cytoplasmic fractions were collected and analyzed through western blot analysis.

### 2.15. Statistical Analysis

The SAS software package (SAS, Cary, NC, USA) was used to analyze data. Mean between-group values were compared using Fisher's exact tests. Bar charts are presented as the mean ± SD. A statistical analysis of mean values was carried out using analysis of variance (ANOVA) in SPSS software (SPSS, Chicago, IL, USA). Differences were considered significant at *P < 0.05.

## 3. Results

### 3.1. Effects of SMEE on Liver Pathological Changes and *α*-SMA Expression Induced by DMN in a Rat Model

**Figure 1:**
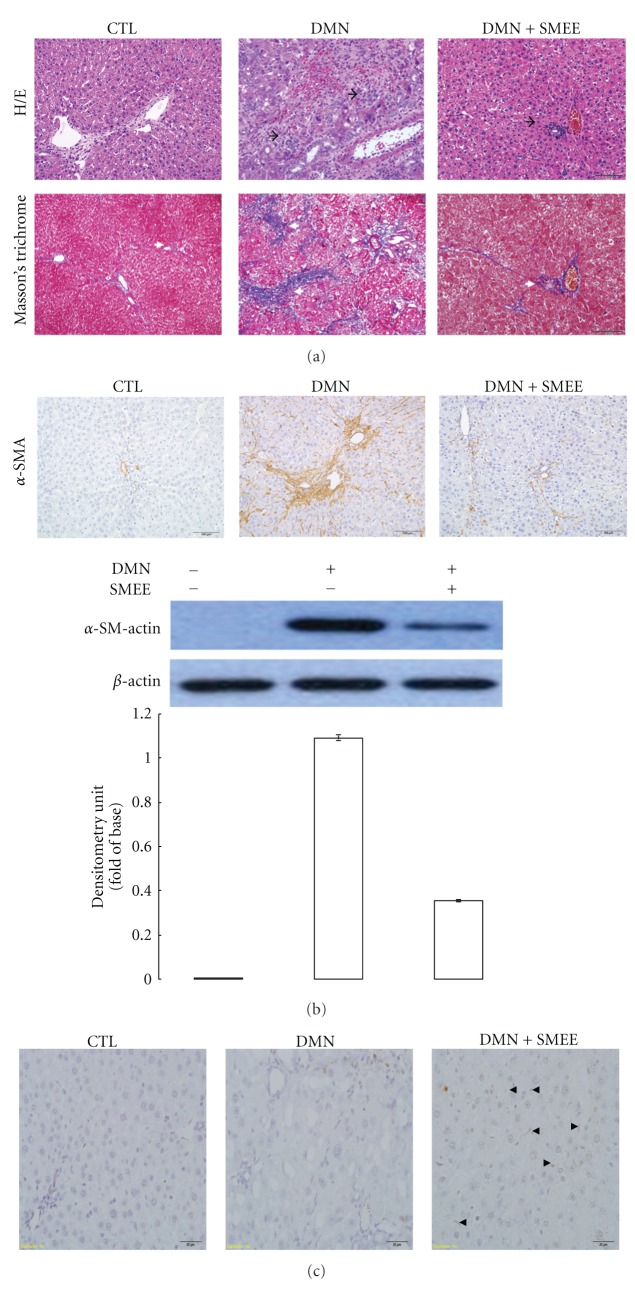
(a) Histologic examination of rat liver by H&E staining and Masson's trichrome staining. (b) *α*-SM-actin expression was presented by immunohistochemical staining. Original magnification: 400x. The intensity of the signals was quantitated by Syngene software and normalized with respect to *β*-actin used as internal controls. The quantitative results were demonstrated as a bar chart. (c) TUNEL assays for detection of apoptotic cells in liver tissues (400x magnification). The arrows indicated TUNEL-positive signals which were shown in brown color.

Piles of evidenced demonstrated that DMN could induce activate-quiescent hepatic stellate cells into proliferating myofibroblast-like cells which result in liver fibrosis. To validate the protective effect of SMEE against hepatic fibrosis *in vivo*, we had examined the histological changes in rat liver tissues. As indicated in H/E staining, the liver of normal control group showed intact lobular architecture with central veins and radiating hepatic cords while DMN application resulted in severe hepatic damage, which manifested as obvious sinusoidal congestion, massive necrosis of hepatocytes, and lymphocyte infiltration (black arrows). On the contrary, SMEE treatment significantly alleviated these pathological damages (Figure 1(a), upper panels). Again, the Masson's trichrome stain showed that DMN led to severe liver fibrosis where, large amount of collagen was accumulated compared with control sample as indicated in blue signals. SMEE attenuated the steatosis, fibrosis, and collagen expression (Figure 1(a), lower panels). In addition, a trace of positive signal of *α*-SMA was observed in control rat liver and marked increase of *α*-SMA was determined under DMN exposure via immunohistochemical staining. In the SMEE-treated groups, quite weak *α*-SMA signal was identified, suggesting that SMEE treatment could effectively eliminate the activated HSCs induced by DMN *in vivo* (Figure 1(b)). Next, we further evaluated apoptotic levels in liver tissues under various treatments. As shown in Figure 1(c), quite rare amount of TUNEL-positive apoptotic cells occurred in the control sample while DMN exposure resulted in moderate expression of TUNEL-positive signal in hepatocytes but not in hepatic stellate cells. Conversely, positively stained stellate cells were remarkably increased in DMN/SMEE group compared to the normal liver and DMN groups. These findings suggested that SMEE could induce apoptosis upon HSCs but not hepatocytes in DMN-induced liver fibrosis in rat.

### 3.2. Inhibitory Effect and Cytotoxicity of Tan IIA on Rat HSC-T6 Cells and HepG2 Cells

**Figure 2:**
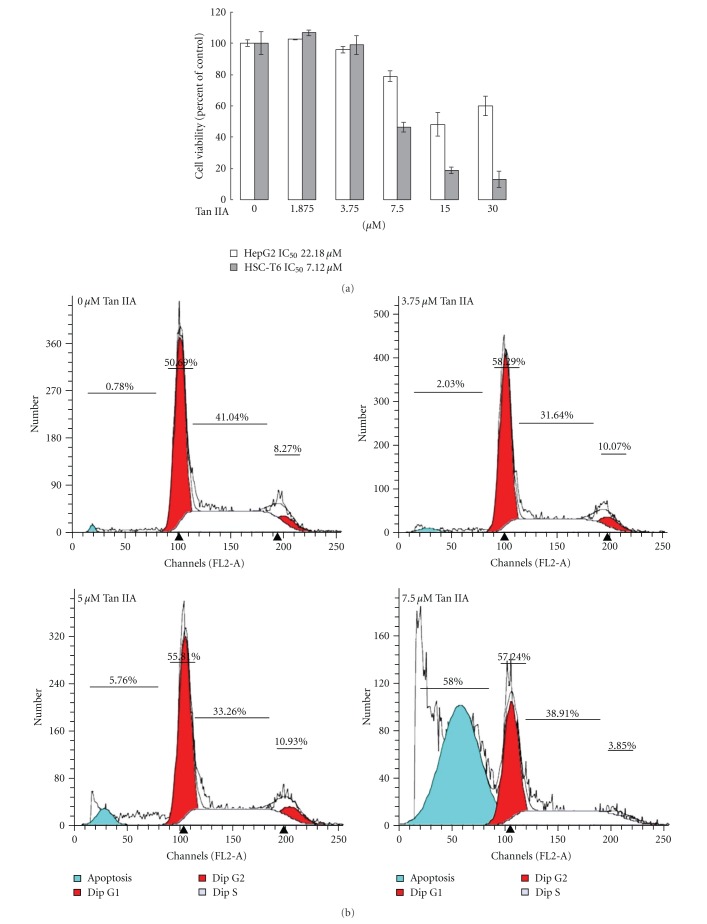

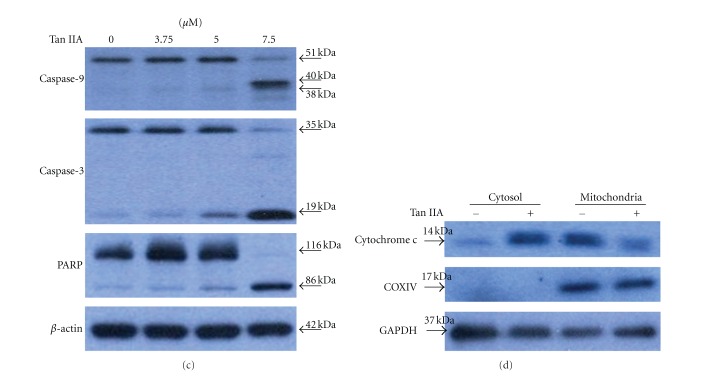
(a) Inhibitory effect of Tan IIA upon HepG2 and HSC-T6 cell viability as measured by the MTT assays. HepG2 cells were indicated in white columns, and HSC-T6 cells were presented in grey columns. Both cells were treated with various concentrations of Tan IIA (*x*-axis). Data are means ± SD of three independent experiments, carried out in triplicate. (b) HSC-T6 cells were treated with 0, 3.75, 5, and 7.5 *μ*M Tan IIA for 24 h. Samples were collected and analyzed by flow cytometry. The peak of the apoptotic phase characterized by the increase in the sub-G1 cell fraction was observed under treatment of 7.5 *μ*M Tan IIA. Ratios of cells in the various phases are represented as a percentage. (c) Treatment of 7.5 *μ*M Tan IIA significantly induced caspase-3, -9 activation and PARP cleavage at 24 h. *β*-actin was used as an internal control. (d) The cytosolic and mitochondria fractions were prepared and subjected to western blot using a specific antibody. COXIV used as a specific mitochondria marker protein was not identified in cytosol fraction to confirm a complete separation of cell fractions. GAPDH was applied as an internal control.

To investigate the pharmaceutical effects of Tan IIA upon HepG2 and HSC-T6 cells, these two cell lines were exposed to 0–30 *μ*M for 24 h, and cell viability was determined by MTT assays. In rat HSC-T6 cells, Tan IIA significantly suppressed cell growth in a dose-dependent manner with a 50% cell growth inhibition value of 7.12 *μ*M while the cell viability of HepG2 cells was not obviously inhibited under Tan IIA application (IC_50_ = 22.18 *μ*M), indicating that Tan IIA could effectively diminish cell viability of activated HSC-T6 cells without causing damage to hepatocytes (Figure 2(a)). Activated HSC-T6 cell death was further evaluated via flow cytometric analysis. As shown in Figure 2(b), only small proportion of the control group of HSC-T6 cells represented in the apoptotic peak and only 8.27% of the normal HSC-T6 cells showed a distribution in G2/M phase. After treatments of 3.75 and 5 *μ*M Tan IIA for 24 h, the cells in the apoptotic and G2/M phase were slightly increased. At a concentration of 7.5 *μ*M Tan IIA for 24 h, the flow cytometric analysis shifted massively toward the apoptotic phase with respective ratios of 58%. We next determined the cleavage of PARP and activation of caspases in Tan IIA-exposed HSC-T6 cells at various concentrations. Correspondingly, the proteolytic cleavage of PARP and active forms of caspase-3 (19 kDa) and caspase-9 (40 kDa) were shown in a dose-dependent manner (Figure 2(c)). Meanwhile, western blotting analysis indicated that Tan IIA induced the release of cytochrome c from mitochondria to cytosol. In our experiment, COXIV was used as a specific mitochondria marker to indicate a complete separation between intracellular fractions (Figure 2(d)). These results suggest that 7.5 *μ*M Tan IIA effectively induces apoptosis of activated HSC-T6 cells through cytochrome c/caspase-associated pathways. We therefore used 7.5 *μ*M Tan IIA in the further studies.

### 3.3. Proteomic Profiling to Tan IIA-Caused Apoptosis in HSC-T6 Cells and Network Analysis

**Figure 3:**
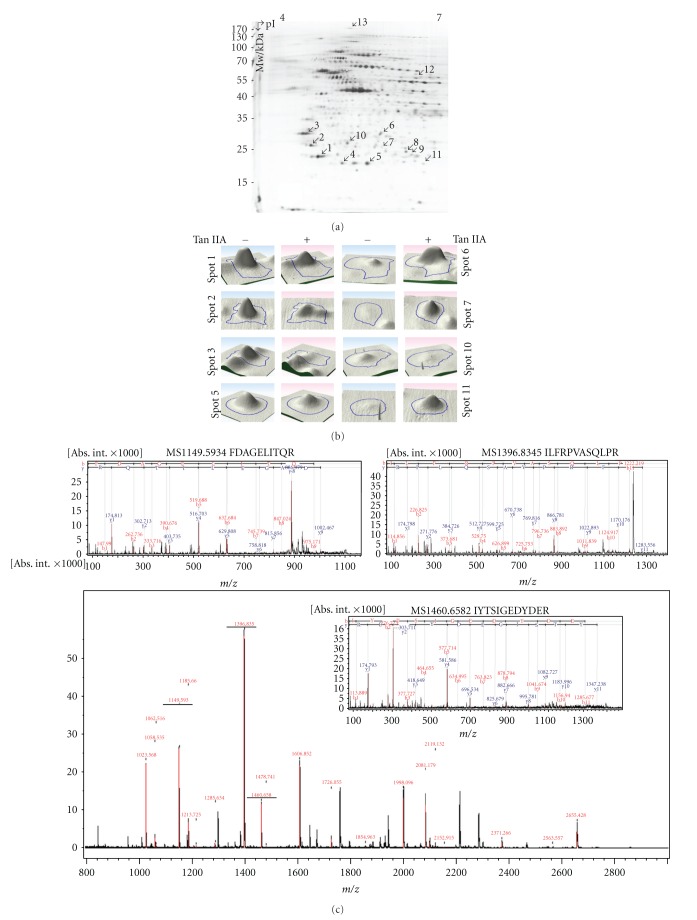

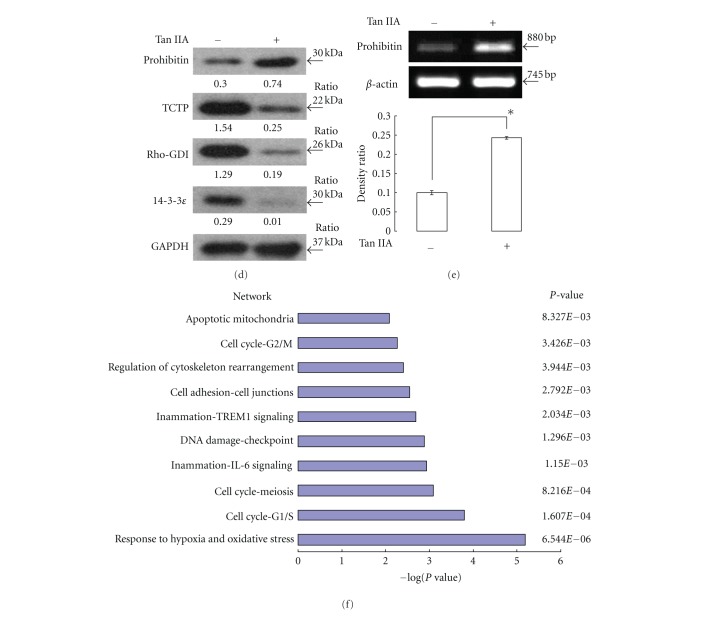
(a) Typical two-dimensional gel electrophoresis (2-DE) pattern of HSC-T6 cells treated with 7.5 *μ*M Tan IIA for 24 h. The protein lysate (300 *μ*g) was focused on a pH 4–7 linear IPG strip before being separated on a 10% polyacrylamide gel. Protein spots with significant changes in intensity are labeled with Arabic numerals. (b) Three-dimensional images of protein spots with up- or downregulated expression. The blue lines delineate the edge of each protein. (c) An MALDI-TOF spectrum of trypsinized prohibitin and the parent ions m/z 1149.5934, 1396.8345, and 1460.6582 were selected for further analysis by an Ultraflex MS/MS operated in the LIFT mode using FlexControl software. The amino acid sequences were unambiguously assigned to the rat prohibitin protein. A sequence was confirmed from the labeled b- and y-ions in the spectrum. (d) Western blot analysis was applied to validate protein changes revealed by 2-DE analysis. GAPDH was used as an internal control, and the relative expression to GAPDH was shown at the bottom. (e) Semiquantitative RT-PCR. Tan IIA treatment upregulated the mRNA expression of prohibitin. *β*-actin was used as an internal control. Each bar represents the mean ± SD calculated from 3 independent experiments. *P < 0.05 compared to the control. (f) Biologic network analyses of differentially expressed proteins using MetaCore mapping tools. Top 10 pathways are ranked based upon *P-*value, and bars represent inverse log of the *P-*value.

To further explore the molecular mechanisms by which Tan IIA induced apoptosis of activated HSC-T6 cells, 2-DE profiles together with MALDI-MS analysis were used to reveal the global protein changes after Tan IIA treatments.  Figure 3(a) demonstrated the representative gel image of control sample visualized by silver staining. In total, 1052 protein spots appeared in the 2-DE maps and a computer-assisted analysis of the respective protein spots revealed 13 protein targets with significant and meaningful changes. Results in all three repeats were consistent and reproducible. Three-dimensional and close-up images of selected proteins were shown to have differential protein expressions (Figure 3(b)). All the 13 proteins were unambiguously identified by MS/MS analyses, and a typical example was shown in Figure 3(c). Prohibitin with a maximum of 26 matching peptides, representing 92% sequence coverage, was characterized. The comprehensive results of the spectrometric analyses and protein functions were summarized in Table 1. To further confirm the changes in protein levels under Tan IIA treatment, a western blot assay was performed. In line with the proteomic results, we detected marked increases in levels of prohibitin compared to the control, whereas TCTP, GDIR1, and 14-3-3*ε* showed remarkable downregulation (Figure 3(d)). Meanwhile, semiquantitative RT-PCR was conducted to determine whether Tan IIA-caused regulation of prohibitin protein was correlated with changes of those messenger RNAs. Our data indicated that mRNA levels of prohibitin were significantly upregulated in Tan IIA-treated group with respect to the control, revealing a good correlation with the protein expression of prohibitin via the western blot analysis (Figure 3(e)). These results indicated that Tan IIA might enhance prohibitin expression via transcriptional and followed translational levels.

To assess the global interaction of the differentially expressed proteins revealed by the proteome analysis and their significance in the mechanisms associated with the antifibrosis effect of Tan IIA, proteins were analyzed by applying the MetaCore analytical tool. As shown in Figure 3(f), the top 10 enrichment networks indicated that proteins differentially expressed after treatment with Tan IIA were primarily involved in the following processes: response to hypoxia and oxidative stress (*P* = 6.544 × 10^−6^), cell cycle-G1/S (*P* = 1.607 × 10^−4^), and cell cycle-meiosis (*P* = 8.216 × 10^−4^).

### 3.4. Knockdown of Prohibitin Attenuates Apoptosis of HSC-T6 Cells under the Treatments of Tan IIA

**Figure 4:**
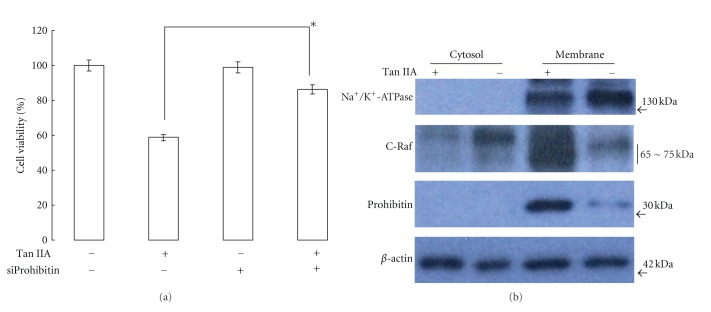
(a) Cell viability was determined with or without RNA interference-mediated silencing of prohibitin under exposure to 7.5 *μ*M Tan IIA. The quantified results were indicated by the bar chart. Results represent the mean±SD of three independent experiments (*P < 0.05). (b) Tan IIA enhanced prohibitin expression and C-raf translocation in HSC-T6 cells. The membrane and cytosolic fractions were prepared and subjected to western blot using a specific antibody. Na^+^/K^+^-ATPase *α*1 used as a specific membrane marker protein was not identified in cytosol fraction to confirm a complete separation of cell fractions. *β*-actin was used as an internal control.

Of the protein targets, prohibitin was significantly upregulated in HSC-T6 cells under Tan IIA treatment and it has been connected to distinct cellular pathways such as apoptosis and cell signaling. Therefore, we next investigated the possible role of prohibitin in modulating the apoptotic pathways induced by Tan IIA at the molecular level. As indicated in Figure 4(a), silence of prohibitin by small interfering RNAs significantly ameliorated cell apoptosis compared to the cells without knockdown of prohibitin in response to 7.5 *μ*M Tan IIA. Meanwhile, no impact on cell viability was found in the prohibitin abrogation group with respect to control sample. This finding suggested that prohibitin should at least partly participate in apoptotic signaling caused by Tan IIA.

### 3.5. Tan IIA Induces Apoptotic Cell Death through C-Raf/Prohibitin Complex Mediated Activation of the MAPK/Bax/Caspase Signaling Pathway

**Figure 5:**
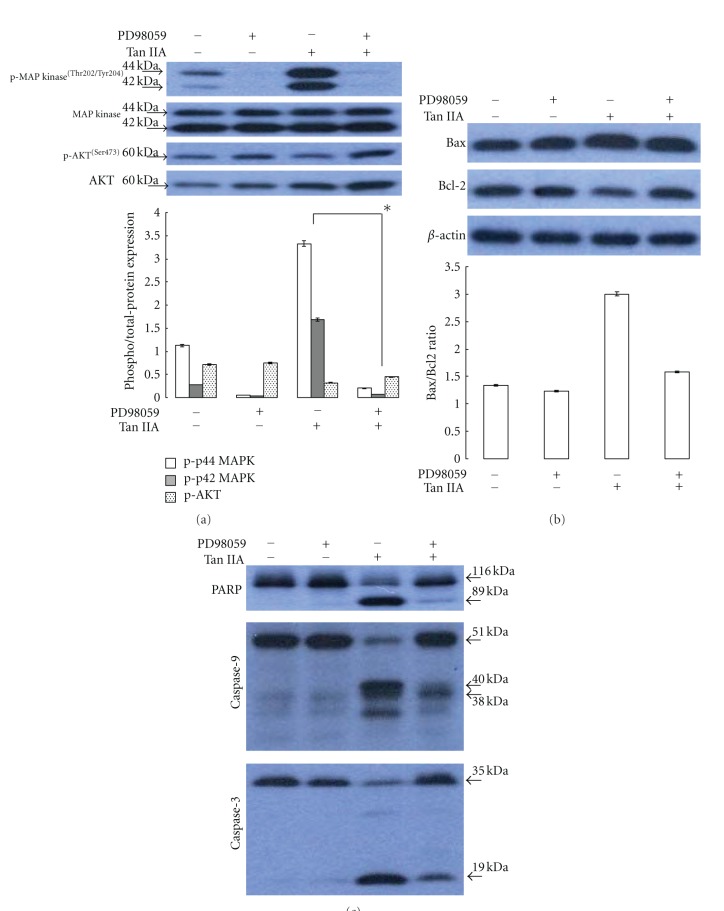
(a) Western blot analysis for phosphorylation and total protein levels of ERK1/2 and AKT with or without PD98059 for 0.5 h. Then the cells were treated with or without Tan IIA for 6 h. The phosphorylation levels of ERK1/2 and AKT were normalized by total protein levels. Results represent the mean ± SD of three independent experiments (*P < 0.05). (b) The protein levels of Bax, Bcl-2, and cytochrome c and (c) cleavage of caspase-3, caspase-9, and PARP with or without treatments of PD98059 and Tan IIA were determined by western blotting assays. Density ratio of Bax over Bcl-2 was measured by densitometer, and GAPDH was used as an internal control. The quantified results were indicated by the bar chart.

Emerging lines of evidence have indicated that prohibitin could bind to C-Raf, which in turn activates MAPK cascades. To further address the signaling events underlying the apoptotic response of HSC-T6 cells after Tan IIA application, we investigated the cellular localization and expression level of C-Raf by a western blot analysis. As indicated in Figure 4(b), on stimulation of Tan IIA, an increase in intracellular translocation of the cytosolic C-Raf protein to the membrane was observed. Prohibitin, on the other hand, showed a predominant localization in the membrane. Herein, Na^+^/K^+^-ATPase was used as a specific membrane marker to demonstrate a complete separation between membrane and cytosol fractions while *β*-actin was used as a loading control. Moreover, activation of MAPK pathway by prohibitin and C-Raf complex might trigger downstream signals which are associated with apoptosis induction. We next estimated whether MAPK family proteins, ERK1/2, contribute to Tan IIA-induced apoptotic cell death. Phosphorylated ERK1/2 (pERK) remarkably increased after 7.5 *μ*M Tan IIA treatment compared to the control, while pretreatment with specific inhibitor PD98059 (50 *μ*M) dramatically inhibited ERK1/2 phosphorylation which was stimulated by Tan IIA. In parallel with this result, Tan IIA-suppressed phosphorylation of AKT was also increased by treatment with PD98059, implying that Tan IIA leads to cell apoptosis through activating ERK1/2 and blocking AKT signaling (Figure 5(a)). Based on our results, Tan IIA might directly induce apoptosis of HSC-T6 cells via activation of ERK1/2 pathway, and we investigated expression levels of mitochondrion-dependent proapoptotic proteins in the presence of ERK1/2 inhibitor. As shown in the Figure 5(b), Tan IIA could upregulate the Bax/bcl-2 ratio accompanied by promotion of cytochrome c expression. Furthermore, suppression of phosphorylation of ERK1/2 by specific inhibitor significantly attenuated the levels of these proteins. As expected, upregulated cleavage of caspase-3, -9, and PARP caused by 7.5 *μ*M Tan IIA administration was concomitantly reduced with PD98059 application (Figure 5(c)).

## 4. Discussion

Various Chinese herbal prescriptions have been widely used for liver protection or therapy of hepatic diseases [29]. In the present study, we investigated the inhibitory effects of SMEE against activated hepatic HSCs *in vivo*. SMEE could effectively repress accumulation of collagen and *α*-SM-actin, therefore, protecting liver from fibrogenesis under DMN stimulation. At the same time, SMEE induced apoptosis of DMN-activated HSCs, which was revealed by TUNEL assays. One of the main natural active compounds in SMEE is Tan IIA which should contribute to the antifibrotic effects. Based on our data, Tan IIA exhibited strong inhibitory effect on viability of HSC-T6 cells whereas the cytotoxicity to HepG2 cells is relatively mild, suggesting that Tan IIA could selectively inhibit activated HSC-T6 without damaging normal hepatic cells. Moreover, we have demonstrated that 7.5 *μ*M Tan IIA induced apoptosis of activated HSC-T6 cells at 24 h by promoting cytochrome c release and activating caspase system as well as cleavage of PARP.

Apoptosis of cell is a multifactorial and complicated process where dynamic changes of a large amount of proteins would be involved [30]. Functional proteomic tool can reveal potential drug targets and address global cellular mechanisms through analyses of differential protein profiles [31]. Our results identified that 11 protein spots displayed significant alterations in intensity after Tan IIA treatment compared to the control. These proteins used as inputs for network analysis were grouped into several categories according to their known functions: oxidative stress, cell cycle regulation, and mitochondria apoptosis. A selected description of proteins that may be critical to induce cell death of HSC-T6 is given as follows.

The first top-rank network is mainly responsible for the response to oxidative stress. Interestingly, several reactive oxygen species- (ROS-) related proteins including PRDX2, PRDX3, and PRDX6 showed dramatic changes in volume under Tan IIA exposure. These proteins belong to the PRDX family which is associated with antioxidant defense especially in hepatic cells. Previous reports have demonstrated that high level of PRDX6 is usually associated with intracellular ROS and could mediate repair of damaged cell membrane through reducing the peroxidized phospholipids [32, 33]. In our study, PRDX3 and PRDX6 were significantly increased in HSC-T6 cells after Tan IIA administration. In addition, DCF fluorescent intensity showed that Tan IIA treatment obviously induced intracellular ROS production compared with the control sample within 6 h (see (Figure 1s) in Supplementary Material available online at doi:10.1155/2012/734987). It is therefore possible that Tan IIA might promote generation of ROS and ultimately result in upregulation of PRDX proteins [34]. If ROS continues, cells would further regulate the expression of proteins involved in apoptosis to lead to cell death. Network analysis indicated that cell cycle regulation also plays a pivotal role in Tan IIA-mediated apoptotic effects. Consistent with the previous study [20], we have demonstrated that Tan IIA treatment could arrest the cells in the G1 phase by flow cytometry assays and induce following apoptosis. These findings led us to consider the function of other proteins in the antifibrotic effects of Tan IIA.

In our study, prohibitin overexpression was observed in Tan IIA-treated cells, which was revealed by proteome, western blotting analysis, and semiquantitative RT-PCR. Prohibitin is an abundant and ubiquitously expressed protein which has been involved in diverse cellular functions and might be considered as a key regulator to integrate cell survival and apoptosis signals [35]. Previous studies indicated that prohibitin deficiency might be associated with cell apoptosis while high levels of prohibitin could promote cell differentiation and suppress cell proliferation [36, 37]. However, the role of prohibitin in cell viability and survival is still controversial. Based on the protein profiles and ROS survey, Tan IIA treatment might trigger oxidative stress which would subsequently induce the prohibitin content of cells as demonstrated in (Figure 2s), where H_2_O_2_ application resulted in 1.5-fold upregulation of prohibitin level. In line with the recent study, prohibitin was identified as a modulator in C-Raf signaling pathway [38], and we also observed that C-Raf was recruited from cytosol to membrane during Tan IIA application. Of note, prohibitin located in the membrane of mitochondria was upregulated in response to Tan IIA exposure. The C-Raf protein thereby was interacted with prohibitin and fully activated. In the presence of Tan IIA, C-Raf and prohibitin complex might activate MEK/ERK signaling pathway. As indicated in our previous research, ERK phosphorylation may contribute to initiate p53/p21-dependent mechanisms, which promote the levels of the proapoptotic Bax protein and inhibit bcl-2 expression on HSC-T6 cells [25]. Here, ERK activation followed by Bax phosphorylation and PARP cleavage were also indicated in the western blotting data. Consistently, pretreatment with PD98059 significantly attenuated Tan IIA-induced ERK1/2 phosphorylation and subsequent Bax activation. Thus, chemical PD98059 finally would suppress phosphorylated Bax/cytochrome c-mediated PARP degradation and apoptosis of HSC-T6. Taken together, upregulated prohibitin is required for the Tan IIA-triggered apoptosis by activating C-Raf/ERK/Bax/caspase signaling pathway. Activated ERK sequentially diminished bcl-2 protein level leading to cytochrome c release followed by caspase protein activaion and PARP cleavage, an irreversible step toward apoptosis. Meanwhile, prohibitin has also been reported to facilitate crosstalk between Ras/MAPK/ERK and PI3 K/Akt pathways. Accordingly, Tan IIA strongly suppressed phosphorylation of AKT which facilitates cell survival and growth whereas PD98059 treatment could partially resume AKT phosphorylation reduced by Tan IIA. Taken together, these results imply that prohibitin may provide a platform for modulating the antiproliferative signals under Tan IIA treatment.

The C-Raf protein kinase is a pivotal component of the Ras-RAF-MAPK pathway that couples extracellular signaling to the cytoplasmic kinases MEK and ERK, which in turn activate various transcription factors [39]. It was previously indicated that C-Raf activation also depends on displacement of 14-3-3 from the specific domain of C-Raf [40, 41], and binding of 14-3-3 proteins has been considered to inhibit Ras-mediated plasma membrane recruitment of C-Raf. Based on our findings, an obvious reduction in 14-3-3 level was shown after applying Tan IIA, which might be closely related to activation and phosphorylation of C-Raf for large magnitude of cell apoptosis caused by Tan IIA treatment.

 Activated Ras proteins transmit their signals to a Raf/ERK cascade and Ras proteins acting as molecular switches that cycle between active GTP-bound and inactive GDP-bound forms [42]. After treatment with Tan IIA, GDP dissociation inhibitor for the rho proteins (rho GDI) which inhibits the subsequent binding of GTP to the rho proteins dramatically decreased as indicated in 2-DE map. Therefore, active Ras (Ras-GTP) would consequently result in C-Raf activation.

## 5. Conclusion

**Figure 6:**
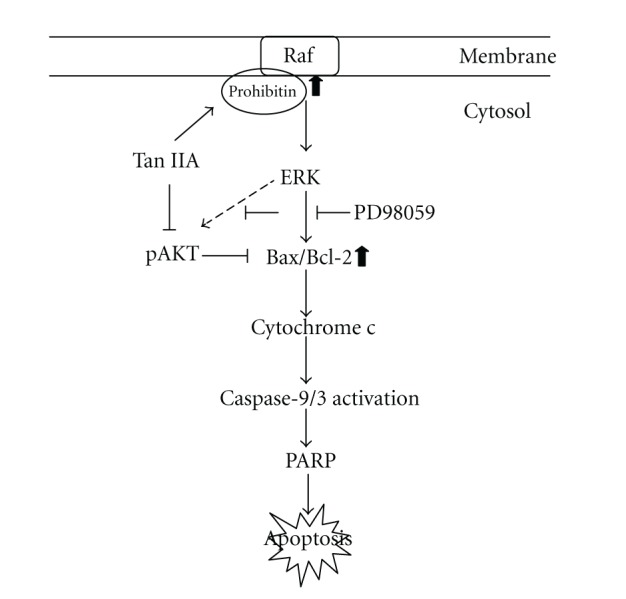
Schematic diagram of Tan IIA-mediated HSC-T6 apoptosis through AKT inhibition and ERK-Bax-caspase-3/9 signaling pathways.

In summary, our results indicated that Tan IIA upregulated ERK pathway which in turn activated Bax via upregulating prohibitin and recruiting C-Raf, while decreasing the expression of 14-3-3 and GDI as well as AKT phosphorylation. MAPKs-induced Bax also resulted in cytochrome c release subsequently leading to caspase proteins-dependent apoptosis (Figure 6). To the best of our knowledge, no published data is available regarding the functional proteome analysis of the inhibitory effect of Tan IIA on apoptosis of HSC-T6 cells. The pivotal target proteins involved in the regulatory mechanism were investigated in the current study.

## Supplementary Material

Supplementary Figure 1S: Evaluation of ROS production using DCF.
Supplementary Figure 2S: Prohibitin expression under H_2_O_2_ stimulation.

## Figures and Tables

**Table 1:** Differentially expressed proteins of hepatic stellate cells follow tanshinone IIA treated.

Spot number	Protein name	Accession number	Mw (kDa)	pI	Coverage (score)	Matched peptide	MS/MS fragment	Ratio^a^	*P* value^b^	Biological function
1	TCTP	P63029	19.56	4.88	40 (97%)	9	^ 6^DLISHDELFSDIYK^19^ (71)	−2.63 ± 0.02	0.01	Involved in calcium binding and microtubule stabilization
2	PSA5	P34064	26.55	4.79	36 (66%)	7	^ 11^GVNTFSPEGR^20^ (25)	−2.18 ± 0.06	0.02	The proteasome has an ATP-dependent proteolytic activity
3	14-3-3E	P62260	29.33	4.63	47 (57%)	7	^ 131^YLAEFATGNDRK^142^ (31)	−3.12 ± 0.05	0.03	Adapter protein implicated in the regulation of a large spectrum of both general and specialized signaling pathways
4	PRDX2	P35704	19.90	5.29	45 (102%)	11	^ 140^QITVNDLPVGR^150^ (26)	2.58 ± 0.04	0.05	Involved in redox regulation of the cell
5	PEBP1	P31044	20.90	5.48	71 (94%)	12	^ 81^FREWHHFLVVNMK^93^ (29)	2.83 ± 0.06	0.03	Binds ATP, opioids, and phosphatidylethanolamine
6	PHB	P67779	29.86	5.57	92 (279%)	26	^ 94^ILFRPVASQLPR^105^ (80) ^106^IYTSIGEDYDER^117^ (44) ^134^FDAGELITQR^143^ (50)	3.25 ± 0.08	0.01	Prohibitin inhibits DNA synthesis
7	HSPB1	P42930	22.94	6.12	62 (118%)	13	^ 29^LFDQAFGVPR^38^ (52)	3.92 ± 0.10	0.03	Involved in stress resistance and actin organization
8	PRDX6	O35244	24.86	5.64	53 (106%)	12	^ 25^FHDFLGDSWGILFSHPR^41^ (35) ^42^DFTPVCTTELGR^53^ (42) ^133^VVFIFGPDKK^142^ (35)	2.42 ± 0.04	0.04	Involved in redox regulation of the cell
9	PRDX3	Q9Z0V6	28.56	7.14	73 (37%)	8	^ 172^DYGVLLESAGIALR^185^ (26) ^198^HLSVNDLPVGR^208^ (30)	2.28 ± 0.04	0.04	Involved in redox regulation of the cell
10	GDIR1	Q5XI73	23.45	5.12	70 (135%)	15	^ 59^VAVSADPNVPNVIVTR^74^ (34) ^100^QSFVLKEGVEYR^111^ (41)	2.56 ± 0.03	0.04	Regulates the GDP/GTP exchange reaction of the Rho proteins by inhibiting the dissociation of GDP from them, and the subsequent binding of GTP to them
11	ATP5H	P31399	16.08	8.92	59 (90%)	7	^ 33^SWNETFHTR^41^ (28)	3.38 ± 0.08	0.02	Mitochondrial membrane ATP synthase
12	ALDH2	P11884	56.97	6.63	88 (27%)	15	^ 162^TIPIDGDFFSYTR^174^ (27)	2.42 ± 0.12	0.03	An aldehyde + NAD^+^ + H_2_O = a carboxylate + NADH
13	HYOU1	Q63617	111.45	5.11	66 (17%)	9	^ 439^DAVIYPILVEFTR^451^ (30)	2.82 ± 0.09	0.05	Has a pivotal role in cytoprotective cellular mechanisms triggered by oxygen deprivation.

^a^Ratios indicated the fold changes of protein volume between Tan-IIA-treated and -untreated cells for each specific protein spot. The higher ratios (>2.0) mean the proteins whose expression levels were increased upon treatment of Tan-IIA, while lower ratios (<−2.0) indicate the proteins were downregulated under the exposure to Tan-IIA.

^b^ *P* values were generated by analyzing the gel images using Prodigy SameSpots software. Differences were considered significant at *P* < 0.05.
