# Supplementary material for: Explore the Molecular Mechanism of Apoptosis Induced by Tanshinone IIA on Activated Rat Hepatic Stellate Cells
Source: Evid Based Complement Alternat Med. 2012 Dec 30;2012:734987. doi: 10.1155/2012/734987 (PMC3546466; doi:10.1155/2012/734987)
Supplement: Supplementary file 1 — Supplementary Figure 1S: Evaluation of ROS production using DCF. Supplementary Figure 2S: Prohibitin expression under H2O2 stimulation. [file 734987.f1.pdf]

Materials and methods of supplemental figures

S1. Image analysis for generation of intracellular ROS under Tan IIA application

2×10^4 HSC-T6 cells were seeded in a slide chamber, grown to 60% confluence, and cultured in serum-free Waymouth medium overnight. Cells were then incubated with 7.5 μM Tan IIA for 6 h. Carboxy-H2DCFDA (4 μM, dissolved in PBS) was added to the wells and incubated for 30 min at 37 °C. To terminate the reaction, the cells were washed with PBS twice. Next, 500 μL culture medium was added to each well and incubated for 20min at 37 °C. The cells were observed and photographed using a fluorescent microscope (Olympus BX51) under DP72 PhotoImage system.

S2. Detection the prohibitin expression level in the presence of ROS

1×10^6 HSC-T6 cells were treated with 500 μM H_2O_2 for 1.5 h and the cells were collected for detection of prohibitin protein level by Western blotting analysis.

Legends of supplemental figures

S1. Cells were incubated with 7.5μM Tanshinone IIA for 6 h and the DCF fluorescence was observed under fluorescence microscope.

S2. Prohibitin levels with or without H_2O_2 were assessed by a Western blot analysis and β-actin was used as an internal control. Density ration of prohibitin over β-actin was measured by densitometer. The quantified results were indicated by the bar chart. *p<0.05, compared to the control.

Figure 1S

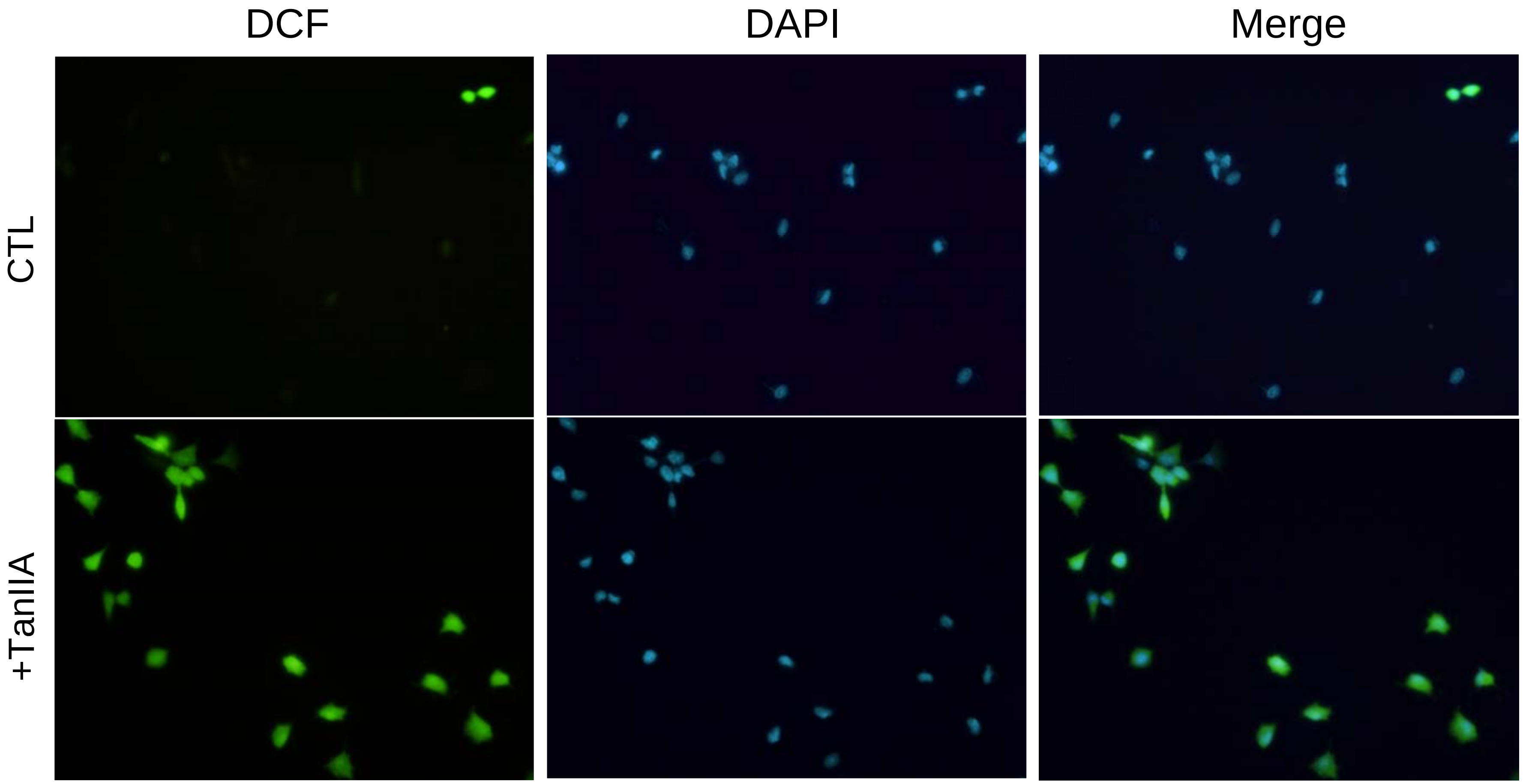

Figure 2S

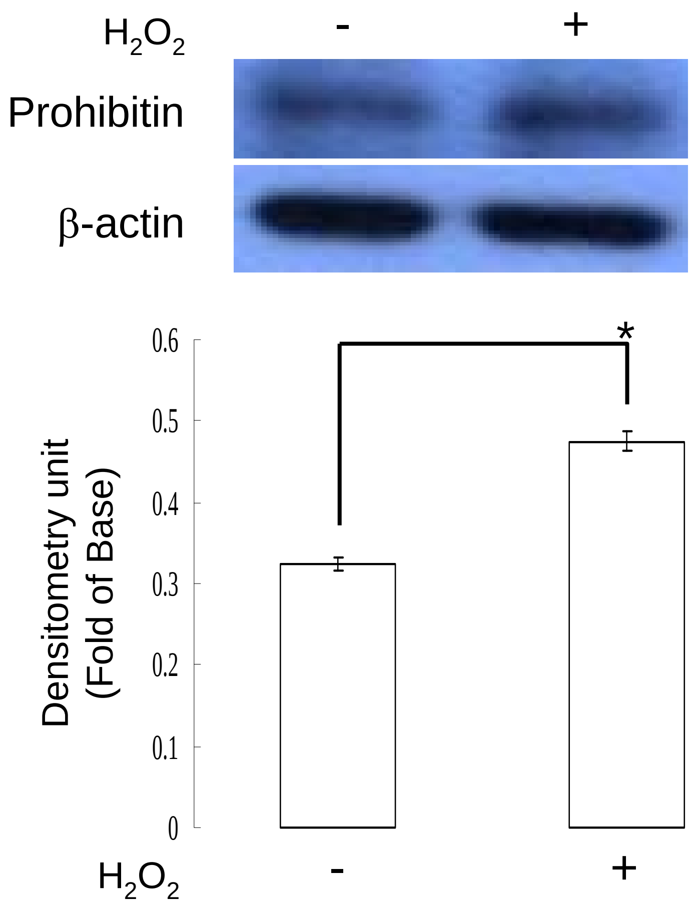
H_2O_2
-
+
Prohibitin
β-actin
*
0.6
0.5
0.4
0.3
0.2
0.1
0
Densitometry unit
(Fold of Base)
H_2O_2
-
+
